# Excessive vincristine exposure in a child being treated for acute lymphoblastic leukaemia with underlying Dubin–Johnson syndrome: a case report

**DOI:** 10.1007/s00280-023-04565-0

**Published:** 2023-07-15

**Authors:** Shelby Barnett, Aye Chan Nyein, Martin Galler, David Jamieson, Michelle Davies, Philip Connor, Gareth J. Veal

**Affiliations:** 1 Newcastle University Centre for Cancer, Translational and Clinical Research Institute, Paul O’Gorman Building, Newcastle upon Tyne, NE2 4HH UK; 2 The Noah’s Ark Children’s Hospital for Wales, Cardiff, UK

**Keywords:** Dubin–Johnson syndrome, ABCC2 gene, Acute lymphoblastic leukaemia, Vincristine

## Abstract

**Background:**

Dubin–Johnson syndrome is a rare benign autosomal recessive condition that causes an isolated increase of conjugated bilirubin in the serum. Impaired biliary excretion is due to mutation in the multiple drug-resistance protein 2 gene (MRP2).

**Case presentation:**

We describe the case of a 4-year-old girl being treated for acute lymphoblastic leukaemia who had a history of conjugated hyperbilirubinaemia and persistently elevated bilirubin levels on initiation of chemotherapy. During treatment for leukaemia, she was diagnosed with Dubin–Johnson syndrome for the underlying condition. Following administration of vincristine at the recommended dose of 1.5 mg/m^2^, an abnormally high vincristine exposure was observed (AUC > 200 µg/L*h), approximately 3 times higher than previously reported exposures in a comparable clinical setting. Vincristine dose reductions were applied on subsequent cycles of treatment and resulted in markedly reduced drug exposures, within the normal target range.

**Conclusion:**

This case provided a rare opportunity to assess the impact of MRP2 mutations associated with Dubin–Johnson syndrome on the pharmacokinetics of vincristine and strongly indicates that a marked dose reduction should be recommended. Clinicians should be made aware of the potential for altered drug disposition for agents such as vincristine in patients with this rare genetic condition.

## Introduction

Acute Lymphoblastic Leukaemia (ALL) is a cancer of lymphoid cells characterised by the unregulated proliferation of aberrant immature lymphocytes. ALL commonly progress rapidly and can be fatal within weeks or months if left untreated. It is the most common form of leukaemia found in children, accounting for approximately 25% of all childhood cancers [1]. Treatment typically consists of chemotherapy aiming to achieve complete remission and cure. ALL chemotherapy consists of treatment phases: induction; consolidation; intensification; and maintenance (post-consolidation).The widely used anticancer drug vincristine plays an important role in all phases [2].

Vincristine is a widely used and effective anticancer drug, but its use is associated with major limiting side effects including peripheral neuropathy, bone marrow suppression, mucositis and hepatotoxicity [3]. The biliary system is the main route of elimination of vincristine and hepatotoxicity may present with deranged liver enzymes and hepatomegaly. In patients with cholestasis, dose reduction of vincristine is recommended for patients due to a reduced drug clearance [4].

Dubin–Johnson syndrome (DJS) is a rare genetic disorder first reported well over 50 years ago [5]. It is a benign autosomal recessive condition that causes an isolated increase of conjugated bilirubin in the serum [6]. While usually presenting in adolescence, DJS can cause cholestasis and hepatomegaly in neonates, but is rarely diagnosed at this age [7]. The classic feature of conjugated hyperbilirubinaemia is a result of defective endogenous and exogenous transfer of anionic conjugates from hepatocytes into bile. This impaired biliary excretion is due to mutation in the multiple drug-resistance protein 2 gene (MRP2; ABCC2), located on chromosome 10 [8]. No treatment is required and prognosis is generally good, with most individuals being asymptomatic and having normal lifespans.

## Case report

A 4-year-old girl was diagnosed with ALL after presenting with generalised malaise, leg pain and fever lasting a month of duration. She was born at term with uneventful pregnancy and received approximately one day of phototherapy on day 3 of life to treat newborn jaundice. Her highest bilirubin level reported at this time was 337 µM, with a conjugated bilirubin concentration of 37 µM (normal ranges: bilirubin 3.4–20.5 µM; conjugated bilirubin 0.06–0.48 µM) [9], and she was discharged in good medical condition. At one month of life, the patient again presented with prolonged neonatal jaundice and suspected sepsis. She was treated with intravenous antibiotics and septic work-up was carried out, identifying her blood group as O RhD positive, with a negative direct Coombs test and normal glucose-6-phosphate dehydrogenase (G6PD), galactose-1-phosphate uridyltransferase (GALT), alpha-1 antitrypsin (AAT) and coagulation profile. An incidental finding of transient neutropenia on a blood film was discussed with the local haematology consultant and the case was also reviewed by a superregional liver unit. She was initially followed up regularly at the general paediatric clinic and liver unit, but no definitive diagnosis was made for hyperbilirubinaemia.

At 4 years of age, the patient presented with episodic bilateral lower leg pain, which was reviewed by the trauma and orthopaedic team and assessed as likely growing pain. Following discharge she was then managed by the paediatric team for generalised malaise and fever lasting for a month of duration. A routine investigation was carried out and 44% blasts CD10/19/34 positive were seen on her initial blood film, with a bilirubin level of 41 µM and normal alkaline phosphatase and aspartate transaminase (AST) levels. At this point, she had a 3 cm enlarged liver with smooth texture but no spleenic enlargement, and bilateral cervical lymphadenopathy. A diagnosis of ALL was made on peripheral blood and bone marrow examination and ETV6-RUNX1 gene rearrangement was detected by fluorescent in situ hybridisation, indicating a good prognosis.

Treatment utilising the UKALL 2011 chemotherapy protocol was initiated with standard 3 drug induction of dexamethasone, vincristine and PEG-asparaginase. The patient initially received a full vincristine dose of 1.5 mg/m^2^. This initial treatment was conducted without any measurement of patient drug concentrations or therapeutic drug monitoring. Deranged liver tests are commonly seen in ALL, potentially related to ALL infiltrating the liver, with the abnormal liver tests often resolving rapidly. However, this was not the case in this patient. Her bilirubin level before the first dose of vincristine was 68 µM, with elevated AST and alanine transaminase (ALT) levels of 52 and 32 U/L, respectively. Coagulation screen prothrombin time was normal. The patient had parainfluenza and Covid-19 during this admission, with deranged liver function test and hepatomegaly, and received 50% of the second dose of vincristine. Both bilirubin and ALT increased and an abdominal ultrasound showed hepatomegaly with diffusely echogenic parenchyma. During this time her blood white cell count dropped and serial bone marrow examination showed a rapid early response of the leukaemia. This suggested that initial control of the leukaemia had been achieved but the liver tests remained deranged. Fat-soluble vitamin supplementation and ursodeoxycholic acid treatment was commenced and the case was reviewed by the surgical team, but no surgical intervention was deemed necessary. Samples for genetic blood tests were obtained as part of a hyperbilirubinaemia work-up and analysed by next generation sequencing (NGS), based on the Genomics England gene panel for cholestasis. Serial ultrasound scans of the abdomen were undertaken during the induction chemotherapy period, with the last ultrasound scan showing diffusely enlarged liver with slight echogenicity. No focal lesion was found with patent hepatic vein and no portal hypertension noted. The patient received 50% doses of vincristine through the induction phase of treatment but two doses were omitted due to deranged liver enzymes.

A diagnosis of DJS (OMIM 237500 with ABCC2 gene), was confirmed through genetic testing 7 weeks after the initiation of chemotherapy and the patient was reviewed by the genetics team. The patient was determined to be homozygous for a rare intronic G to A transition immediately 3’ of the last coding nucleotide of exon 3 (rs533334893), believed to result in aberrant splicing and loss of ABCC2 function. Following discussion of the case at a multidisciplinary team meeting, it was decided to adopt a therapeutic drug monitoring (TDM) approach to vincristine treatment and the patient was registered on the national TDM study (ISCRTN 10139334). The first TDM cycle was conducted following administration of a full vincristine dose of 1.5 mg/m^2^ as the patient no longer had elevated bilirubin levels at this point in treatment. Further dosing of vincristine in the patient was determined based on drug tolerability alongside an evaluation of drug exposure as described below. The patient experienced hypertension during her induction chemotherapy which was well controlled with oral antihypertensive medications. At the end of induction (week 5) bone marrow aspirate examination showed morphological remission and low levels of measurable residual disease by molecular methods (quantitative polymerase chain reaction of disease specific Immunoglobulin and T cell receptor gene rearrangements). Prior to initiation of the delayed intensification phase at week 20 of treatment, the patient had a bone marrow aspirate which was negative for measurable residual disease.

## Pharmacokinetic analysis

Blood samples were collected at 0.5, 1, 4, 6 and 24 h post vincristine bolus administration and immediately spun at 1200 g for 5 min to obtain plasma. Plasma samples were then shipped overnight on dry ice to the Newcastle Cancer Centre Pharmacology Group for pharmacokinetic analysis. Samples were extracted and analyzed using a validated liquid chromatography-mass spectrometry (LC–MS) assay, as previously described [10]. Non-compartmental pharmacokinetic analysis of vincristine plasma concentrations was performed using WinNonlin version 8.1 (Certara, Princeton, NJ, USA).

**Fig. 1:**
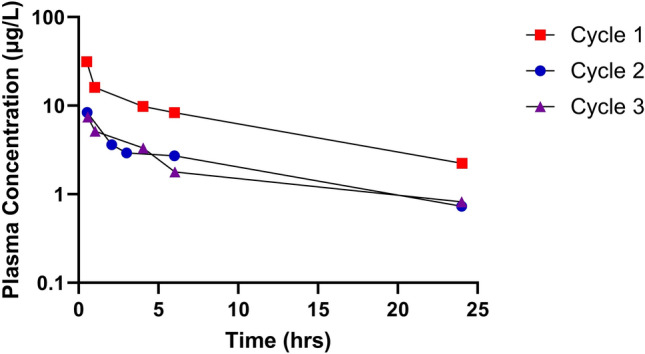
Plasma concentrations of vincristine determined following intravenous drug administrations at doses of 1.5 mg/m^2^ (cycle 1) and 1.0 mg/m^2^ (cycle 2 and 3)

On the first cycle of TDM, the patient received the full standard vincristine dose of 1.3 mg (1.5 mg/m^2^). Pharmacokinetic analysis on this cycle showed that the patient achieved a vincristine area under the plasma concentration–time curve (AUC) of 202 µg/L*h (Table 1). This compares to median AUC values of 78 µg/L*h and 68.1 µg/L*h reported in two recently published studies, incorporating data from close to 200 patients being treated on a comparable dosing regimen [11, 12]. The vincristine exposure observed was markedly higher than the recommended therapeutic window of 50–100 µg/L*h [11], therefore, a dose reduction was suggested. On the second and third cycles of TDM a 33% dose reduction (1 mg/m^2^) was applied and the patient received vincristine doses of 0.8 mg and 0.9 mg, respectively. This reduction in dose resulted in a reduction of vincristine AUC to 64.5 µg/L*h on cycle 2 and 57.5 µg/L*h on cycle 3. Figure 1 shows the pharmacokinetic profiles observed on these three cycles of treatment when vincristine plasma concentrations were determined.

**Table 1:** Vincristine pharmacokinetic parameters observed with full and reduced dosing regimens across three cycles of treatment

Cycle	Dose (mg)	AUC (µg/L*h)	C_max_ (µg/L)	CL (L/h)	Vz (L)
1	1.3	202	31.4	6.4	87
2	0.8	64.5	8.4	12.4	177
3	0.9	57.5	7.4	15.6	218

*AUC* area under the plasma concentration–time curve, *C*_*max*_ maximum plasma concentration, *CL* clearance, Vz apparent volume of distribution

## Discussion

In relation to the diagnosis of DJS in this patient, the presence of cholestasis and hyperbilirubinaemia in infancy could have alerted the physician to the possibility of DJS in the first few weeks of life. Such an early diagnosis would help to differentiate between DJS and other conditions, which may be associated with commonly observed hallmarks of the disease, potentially avoiding unnecessary hospital visits and invasive and time-consuming hospital tests later in life.

An early diagnosis of DJS is also beneficial in terms of considering potential complications of any drug treatment that the patient may require. In this respect, the case study described provided a unique opportunity to assess the impact of mutations in MRP2 associated with DJS, on the pharmacokinetics of the widely used anticancer drug vincristine. The patient initially received a full dose of vincristine, with a 50% dose reduction instigated early in treatment, but multiple doses of drug had to be omitted due to issues with drug tolerability. Utilising a TDM approach to treatment it was observed that the patient had an approximately threefold higher drug exposure than comparable patients included in recently published studies, at the standard vincristine dose of 1.5 mg/m^2^. Most notably, a very low vincristine clearance (CL) value was observed on cycle 1 of treatment, following a full vincristine dose of 1.5 mg/m^2^, presumably associated with saturation of drug clearance processes in this clinical scenario. These data observed with vincristine were in contrast to pharmacokinetic data generated in the same patient for doxorubicin, which was also monitored through the TDM programme. Doxorubicin was administered at full dose and the plasma levels observed were in the expected range for a childhood cancer patient population. In the broader context of pharmacogenomics, while the apparent loss of ABCC2 function resulting in increased exposure to vincristine is indicative of a central role in the drug’s elimination, neither CNS toxicity or marked peripheral neuropathy were noted, indicative of a minimal role for ABCC2 in protection against vincristine induced neurotoxicity. Reported pharmacogenomic studies of ABCC2 SNPs and vincristine induce neurotoxicity are sparse, though a predicted splice variant SNP (rs3740066) and ABCC2 haplotypes appear to be protective against low grade vincristine induced neurotoxicity in paediatric ALL, but are not associated with severe toxicity [13].

## Conclusion

An increased awareness of the symptoms of DJS in neonates would be beneficial in helping to avoid misdiagnosis in cases such as the patient reported in the current study. We recommend use of NGS panels to assist diagnosis in this rare disorder. Furthermore, we hope this article provides information to clinicians of the potential for altered drug disposition for agents such as vincristine, where a reduced dosage in patients with DJS is clearly warranted based on the data presented.

## Data Availability

The clinical and laboratory data supporting this study’s findings are available from the corresponding author upon special request. The datasets generated during the current study are not publicly available for ethical reasons per local guidelines.

## References

[1] Kakaje A (2020). Rates and trends of childhood acute lymphoblastic leukaemia: an epidemiology study. Sci Rep.

[2] Inaba H, Mullighan CG (2020). Pediatric acute lymphoblastic leukemia. Haematologica.

[3] Gidding CE (1999). Vincristine revisited. Crit Rev Oncol Hematol.

[4] Van den Berg HW (1982). The pharmacokinetics of vincristine in man: reduced drug clearance associated with raised serum alkaline phosphatase and dose-limited elimination. Cancer Chemother Pharmacol.

[5] Dubin IN, Johnson FB (1954). Chronic idiopathic jaundice with unidentified pigment in liver cells; a new clinicopathologic entity with a report of 12 cases. Medicine.

[6] Morais MB, Machado MV (2022). Benign inheritable disorders of bilirubin metabolism manifested by conjugated hyperbilirubinemia-a narrative review. United Eur Gastroenterol J.

[7] Lee JH (2006). Neonatal Dubin-Johnson syndrome: long-term follow-up and MRP2 mutations study. Pediatr Res.

[8] Kartenbeck J (1996). Absence of the canalicular isoform of the MRP gene-encoded conjugate export pump from the hepatocytes in Dubin-Johnson syndrome. Hepatology.

[9] https://www.nice.org.uk/guidance/cg98. Accessed 1 May 2023

[10] Israels T (2010). Malnourished Malawian patients presenting with large Wilms tumours have a decreased vincristine clearance rate. Eur J Cancer.

[11] Barnett S (2022). Vincristine dosing, drug exposure and therapeutic drug monitoring in neonate and infant cancer patients. Eur J Cancer.

[12] Skolnik J (2021). Toxicity and pharmacokinetics of actinomycin-D and vincristine in children and adolescents: Children's Oncology Group Study ADVL06B1. Cancer Chemother Pharmacol.

[13] Lopez-Lopez E (2016). Vincristine pharmacokinetics pathway and neurotoxicity during early phases of treatment in pediatric acute lymphoblastic leukemia. Pharmacogenomics.

